# Differential effects of silencing crustacean hyperglycemic hormone gene expression on the metabolic profiles of the muscle and hepatopancreas in the crayfish *Procambarus clarkii*

**DOI:** 10.1371/journal.pone.0172557

**Published:** 2017-02-16

**Authors:** Wenfeng Li, Kuo-Hsun Chiu, Yi-Chun Tien, Shih-Fu Tsai, Li-Jane Shih, Chien-Hsun Lee, Jean-Yves Toullec, Chi-Ying Lee

**Affiliations:** 1 Department of Biology, National Changhua University of Education, Changhua, Taiwan; 2 Department of Aquaculture, National Kaohsiung Marine University, Kaohsiung, Taiwan; 3 Department of Medical Laboratory, Taoyuan Armed Forces General Hospital, Taoyuan, Taiwan; 4 Sorbonne Universités, UPMC Université Paris 06, UMR 7144 CNRS, Equipe ABICE, Station Biologique de Roscoff, Roscoff, France; 5 CNRS, UMR 7144, Adaptation et Diversité en Milieu Marin, Station Biologique de Roscoff, Roscoff, France; International Centre for Genetic Engineering and Biotechnology, ITALY

## Abstract

In order to functionally characterize the metabolic roles of crustacean hyperglycemic hormone (CHH), gene expression of CHH in the crayfish (*Procambarus clarkii*) was knocked down by *in vivo* injection of CHH double-stranded RNA (dsRNA), followed by metabolomic analysis of 2 CHH target tissues (the muscle and hepatopancreas) using nuclear magnetic resonance spectroscopy. Compared to the levels in untreated and saline-injected (SAI) animals, levels of CHH transcript, but not those of molt-inhibiting hormone (a CHH-family peptide), in the eyestalk ganglia of CHH dsRNA-injected (DSI) animals were significantly decreased at 24, 48, and 72 hour post injection (hpi), with concomitant changes in levels of CHH peptide in the sinus gland (a neurohemal organ) and hemolymph. Green fluorescence protein (GFP) dsRNA failed to affect levels of CHH transcript in the eyestalk ganglia of GFP DSI animals. Number of metabolites whose levels were significantly changed by CHH dsRNA was 149 and 181 in the muscle and 24 and 12 in the hepatopancreas, at 24 and 48 hpi, respectively. Principal component analysis of these metabolites show that metabolic effects of silencing CHH gene expression were more pronounced in the muscle (with the cluster of CHH DSI group clearly being separated from that of SAI group at 24 hpi) than in the hepatopancreas. Moreover, pathway analysis of the metabolites closely related to carbohydrate and energy metabolism indicate that, for CHH DSI animals at 24 hpi, metabolic profile of the muscle was characterized by reduced synthesis of NAD^+^ and adenine ribonucleotides, diminished levels of ATP, lower rate of utilization of carbohydrates through glycolysis, and a partially rescued TCA cycle, whereas that of the hepatopancreas by unaffected levels of ATP, lower rate of utilization of carbohydrates, and increased levels of ketone bodies. The combined results of metabolic changes in response to silenced CHH gene expression reveal that metabolic functions of CHH on the muscle and hepatopancreas are more diverse than previously thought and are differential between the two tissues.

## Introduction

Crustacean eyestalks contain a neuroendocrine tissue, the X-organ/sinus gland complex, which synthesizes and secrets several neurohormones that are implicated in regulating a wide variety of physiological functions, including metabolism, reproduction, growth, ionic balance, and color changes. The wide array of biological processes that are under the hormonal control of the eyestalk factors highlights the importance of the XO/SG complex in crustacean physiology [1–3]. Among these neuropeptides, crustacean hyperglycemic hormone (CHH) is the prototypic member of the CHH family, which also includes molt-inhibiting hormone (MIH), vitellogenesis-inhibiting hormone (VIH), and mandibular organ-inhibiting hormone (MOIH), and insect ion transport peptide (ITP) [4, 5]. CHH, a 72- or 73-residue polypeptide, is characterized by 3 disulfide bridges formed by 6 highly conserved cysteine residues and N- and C-terminally blocked, with the C-terminal amide being critical for its hyperglycemic activity [6–10]. Molecular characterizations of CHH precursor indicated that the precursor protein consists of a signal peptide, a CHH precursor-related peptide, and a mature CHH peptide [11].

Functionally, the best characterized roles for CHH is its effects on carbohydrate metabolism [12] and CHH is considered a stress hormone that elicits stress-induced hyperglycemia [13–17]. The majority of the data concerned the effects of CHH on glycogen metabolism. Thus, early *in vivo* and *in vitro* studies have shown that CHH preparations (*i*.*e*., eyestalk or sinus gland extracts) rapidly mobilized glycogen stores in the hepatopancreas and muscle of *Orconectes limosus* and *Uca pugilator*, resulting in decreased levels of tissue glycogen or increased levels of glucose release from the tissues [18–22], and that activity of glycogen synthase was inhibited by CHH preparations (*i*.*e*., sinus gland extracts) and increased by eyestalk ablation [20]. A more recent study showed that transcript levels of glycogen synthase were decreased and those of glycogen phosphorylase increased in hepatopancreatic tissues of *Marsupenaeus japonicus* treated *in vitro* with recombinant CHH [23]. Although most early studies employed tissue extracts (eyestalk ganglia or sinus glands) rather than purified CHH, the combined data are consistent with the notion that CHH mobilizes tissue glycogen store through regulating activity and amount of enzymes in glycogen metabolism. Specific CHH binding sites have been characterized in multiple tissues (including the hepatopancreas and muscle) [24–27].

Surprisingly, with the exception of regulation of glycogen metabolism as just mentioned, evidence for other metabolic effects of CHH was rather limited. Again, relevant data were those obtained from early studies. Thus, it has been proposed that CHH stimulates glycolysis, based on observations that hypoxia increased glucose and lactate levels in the hemolymph of intact animals (*Carcinus maenas* and *Orconectes limosus*), but not in eyestalk-ablated animals; the hypoxia-increased hemolymph levels of glucose and lactate were however observed in eyestalk-ablated animals receiving injection of CHH [28]. The authors considered that these data indicated that CHH stimulates glycolytic flux by increasing the availability of glycolysis substrate (glucose) through its stimulatory effect on glycogen mobilization, and hence leading to elevated levels of lactate [28, 29].

During the past 2 decades or so, much more research effort was directed towards molecular and biochemical characterization of CHH-family peptides and their encoding gene in both crustacean and non-crustacean species [2, 3, 4, 5, 11, 30, 31, 32], while the regulatory function of its prototypic member is still poorly characterized. Thus, the aim of the present study was to elucidate in a more comprehensive manner the regulatory effects of CHH on the metabolism of its target tissues. A double-stranded RNA (dsRNA)-based gene silencing method was developed and validated for specific and effective silencing of CHH gene expression in the eyestalk ganglia, with concomitant decreases in CHH levels in the sinus gland and hemolymph. Subsequently, metabolites in the tissues (the hepatopancreas and muscle) harvested from saline-injected and CHH dsRNA-injected animals were identified and quantified using nuclear magnetic resonance spectroscopy. The resulting data clearly indicate that CHH has more diverse regulatory effects on tissue metabolism than previously known and exerts its metabolic effects in tissue-specific and complementary manners.

## Materials and methods

### Experimental animals and procedures

Experimental protocols involving animals employed by the present study were approved by the review committee of National Changhua University of Education (Permit number: NCUE-103320001), in full accordance with the recommendations (Guidelines for Management and Use of Experimental Animals) set by the Council of Agriculture, Taiwan. Animals (*P*. *clarkii*) used in the present study were trapped and supplied by local fisherman from Xihu River, Miaoli County, Taiwan (24.25° N, 120.45° E), and reared in the laboratory in water tanks with temperature held at 24 ± 1°C and a 12L/12D photoperiod regime [16]. Animals used in the present study were all in intermolt stage [33] with body weights ranging from 5 to 6 grams.

Experimental procedures described below were always carried out in the morning (9–11 a.m.) in order to reduce possible interference due to circadian changes in CHH and hemolymph glucose levels [34, 35]. Animals were separated into 3 groups (6 animals per group): the untreated group, saline-injected (SAI) group, and dsRNA-injected (DSI) group.

Animals in the SAI and DSI groups were intramuscularly injected using an insulin syringe coupled to a 29-gauge needle (Terumo Medical Corp.) with crayfish saline (50μl/animal, 4-(2-hydroxyethyl)-1-piperazineethanesulfonic acid (HEPES)-buffered Van Harreveld saline, pH = 7.4, [16]) and saline containing CHH dsRNA or green fluorescence protein (GFP) dsRNA (50μl/animal, 30μg dsRNA/g body weight), respectively. Hemolymph, eyestalk ganglia, muscle, and hepatopancreas were collected from animals of the untreated group and from animals of the SAI and DSI groups at 24, 48, and 72 h post injection (hpi). Briefly, hemolymph was withdrawn using a syringe coupled to a 26G syringe and processed according to Yeh et al. [36] for quantification of CHH and glucose levels (see below “CHH sandwich enzyme-linked immunosorbent assay (ELISA) and glucose assay”). Sinus glands were completely removed from the eyestalk ganglia, homogenized in HEPES-buffered saline, and centrifuged at 16,000*g* for 30 min at 4°C; the supernatants were stored at -80°C until used for quantification of CHH levels. The rest of the eyestalk ganglia were used for extraction of total RNA (Purescript RNA Isolation Kit, Gentra), which was then treated with RQ1 RNase-free DNase (Promega) according to the supplier′s procedures. For cDNA synthesis, a reverse transcription reaction was performed as previously described [37] and the synthesized cDNA was kept at -20°C until used for quantitative real-time polymerase chain reaction (qPCR) (see below “Semi-quantitative real-time polymerase chain reaction”). Dissected muscle and hepatopancreas were boiled using a hot plate (100°C, 5 min, Corning) and frozen in liquid nitrogen until used for nuclear magnetic resonance (NMR) analysis (see below “Nuclear magnetic resonance analysis of metabolites”).

### Production of CHH and GFP double-strained RNAs (dsRNAs)

Double-stranded RNAs were produced using an *in vitro* transcription reaction driven by T7 promoter and T7 RNA Polymerase (T7 RiboMAX^™^ Express RNAi System, Promega).

For the production of CHH dsRNA, sense and antisense CHH DNA templates each with a T7 promoter sequence were separately amplified by PCR with primers containing T7 promoter sequence. Primer pairs used were: T7-CHH1-352-357-F and CHH1-472-492-R (for sense strand) or CHH1-352-357-F and T7-CHH1-472-492-R (for antisense strand) (S1 Table). A 25-μl PCR reaction contained 5μl 5X Colorless GoTaq Flexi Buffer (Promage), 2.5μl MgCl_2_ (25 mM), 0.5μl dNTP (10mM), 0.5μl forward primer (10mM), 0.5μl reverse primer (10mM), 0.13μl GoTaq DNA polymerase (5U/μl) and 1μl cDNA (eyestalk cDNA synthesized as mentioned in “Experimental animals and procedures”). PCR products of expected size were purified using a commercial kit (PCR Advanced^™^, VIOGENE) and auto-sequenced to confirm their sequences. Using the CHH sense strand and antisense strand as DNA templates, complementary single-stranded RNA was produced *in vitro* using T7 RiboMAX^™^ Express RNAi System (Promega) according to the supplier′s instruction. Resulting sense and antisense RNAs were mixed, heated at 70°C for 10min, and annealed to each other by cooling the mixture to 25°C at a rate of -15°C/hr. The dsRNA mixture was then treated by RQ1 RNase-Free DNase and RNase A Solution (Promega) to remove unwanted DNA and single-stranded RNA, precipitated with isopropanol, air-fried, and re-suspended in nuclease-free water following the supplier′s instruction. The purified dsRNA samples were spectrophotometrically quantified (NanoDrop1000, Thermo), confirmed for integrity using non-denaturing 2% agarose gel electrophoresis (S1 Fig), and kept at -80°C before being used for *in vivo* injection.

Sense and antisense GFP DNA templates each with a T7 promoter sequence were separately amplified by PCR. Briefly, a plasmid containing a GFP insert (L4417, Addgene) was amplified using a primer pair: GFP-F-58-78-P144 and GFP-R-181-201-P144 (S1 Table). The resulting amplified GFP product was purified from the reaction mixture and then used as the template for a second PCR using the PCR primers containing T7 promoter sequence (S1 Table): F-T7-GFP-P140-ds & GFP-R-181-201-P144 (for GFP sense strand) or GFP-F-58-78-P144 & R-t7-gfp-p140-ds (for GFP antisense strand). Subsequently, using the GFP sense strand and antisense strand as DNA templates for *in vitro* transcription reaction, complementary single-stranded RNA was produced and annealed, and the resulting GFP dsRNA mixture was treated and processed as mentioned above for CHH dsRNA.

### CHH sandwich enzyme-linked immunosorbent assay (ELISA) and glucose assay

CHH peptide levels in sinus gland and hemolymph samples were quantified using an established sandwich ELISA with 2 anti-CHH antibodies; specificity of the assay for the crayfish CHH has been demonstrated and reported [38, 39]. Glucose levels in the hemolymph samples were quantified using an established glucose assay (GAGO20-1KT, Sigma). Protocols and reagents for CHH ELISA and glucose assay have been described previously [16, 38, 39].

### Semi-quantitative real-time polymerase chain reaction (qPCR)

A semi-quantitative real-time PCR was used to assess the effects of CHH or GFP dsRNA on the expression of the target gene (*CHH*) and a related gene *MIH* (molt-inhibiting hormone gene, a CHH family-peptide gene) that serves as an off-target gene. Primers used were CHHe2F and SG-R-400-427 for CHH gene [40] and MIH-F and MIH-R for MIH gene (S1 Table). *18S rRNA* (18S ribosomal RNA gene) was used as the reference gene [40], and the primers used for its amplification were 18S-F-1280 (forward) and 18S-R-1360 (reverse) (S1 Table). Briefly, eyestalk ganglia cDNA templates were amplified in a 10-μl reaction using a commercially available kit (LightCycler FastStart DNA Master SYBR Green I, Roche) according to manufacturer′s protocol. Reactions were performed under the following conditions: an initial denaturation (10 min, 95°C), 40 cycles of denaturation (10 s, 95°C), annealing (7 s, 60°C), and extension (14 s, 72°C); the rate of temperature change was 20°C/s.

Amplification efficiency and specificity were validated according to previously described methods [37]. For each of the 3 amplicons (*CHH*, *MIH*, and *18S rRNA*), PCR conditions were optimized to ensure an amplification efficiency greater than 90%. To ensure amplification specificity, melting curve analysis was performed from 65°C to 94°C at the rate of 0.1°C/s after each PCR assay. For each of the 3 amplicons, melting curve analyses showed only a single peak indicating the amplification specificity of the reaction.

The comparative threshold cycle method [41] was used to determine the transcript levels. Threshold cycle number for each PCR was determined using the Second Derivative Maximum algorithm that identifies the first turning point of the fluorescence curve (Light-Cycler software, v. 3.5). Transcript levels of *CHH* or *MIH* were normalized to those of *18S rRNA* and the normalized levels at each time points after injection were expressed relative to the levels of untreated animals (arbitrarily designated as the calibrator), according to previously described equations [37].

### ^1^H Nuclear magnetic resonance (NMR) analysis of metabolites

Tissue metabolites were extracted using an extraction procedure according to Teng et al. [42] with modifications. In brief, tissues (the muscle and hepatopancreas) collected from the animals in different treatment groups (see “Experimental animals and procedures”) were frozen in liquid nitrogen and ground in ceramic mortar using a pestle. Ground tissue powders were added to a centrifuge vial containing cold 50% methanol (250 μl per 0.1g tissue powders), and homogenized at 4°C for 15 min using a tissue homogenizer (Bullet Blender^®^, Next Advance). Resulting homogenate was collected and an aliquot of the homogenate (250 μl) was transferred to a new vial containing an equal volume of 50% methanol, followed by adding 0.5ml of chloroform to the mixture. The mixture was further homogenized for 20 min at 4°C, centrifuged at 12,000g for 15 min at 4°C. The resulting aqueous phase was collected. Extraction solvents were completely removed using a vacuum concentrator (Savant SpeedVac, Thermo Scientific). Samples were then reconstituted in deuterium oxide (D_2_O, Merck) containing 1 mM sodium salt of 3-(trimethylsilyl) propionate-2,2,3,3-d_4_ (TSP) for NMR analysis.

All NMR spectra were acquired at 25°C on a Varian Inova 500 MHz NMR spectrometer (Varian, Inc.). The residual water resonance was pre-saturated using the PRESAT sequence. Typically, 128 transients were summed over a spectral width of 8 kHz into 32,000 data points per sample, and the summed free induction decays (FIDs) were Fourier transformed with exponential line-broadening of 0.3 Hz [43].

### Statistical and principal component analyses of metabolites

The spectra were phased, baseline-corrected, and referenced to TSP (at 0.0 ppm) as well as quantified and qualified using a commercial software package (Chenomx NMR suite 4.6, Chenomx, Inc., Edmonton, Alberta, Canada) with the Chenomx 500-MHz (pH 6–8) library. The spectra data were pre-processed using normalization and scaling to remove possible bias arising due to sample handling and sample variability. Normalization (by sum) was performed in order to minimize possible differences in concentration between samples. In addition, the spectral region from 5.0 to 4.7 ppm containing the residual water resonance was excluded, and all subsequent analysis used centred scaling.

After data pre-processing, a univariate statistical analysis (Student′s t-test) was used to verify the significance of difference in metabolite levels between the CHH dsRNA-injected group and the saline-injected group. p<0.05 was considered to indicate a statistically significant difference. These significantly changed metabolites were further analyzed by heuristic methods of dimension reduction (principal component analysis [PCA]) using commercially available software (SIMCA-P 11.5; Umetrics, Umeå, Sweden), and all subsequent analysis used centred scaling. The number of PCA components was calculated using an auto-fit model in SIMCA-P (Umetrics).

### Statistical analysis of transcript, peptide and glucose levels

Data for CHH transcript and peptide levels and glucose levels were analyzed by one-way analysis of variance followed by Tukey's HSD test or by Student's t-test using computer software (SPSS Manager, SPSS Inc.) and are reported as the mean ± standard deviation.

## Results

### Effects of dsRNA treatments on transcript levels in the eyestalk ganglia

**Fig 1:**
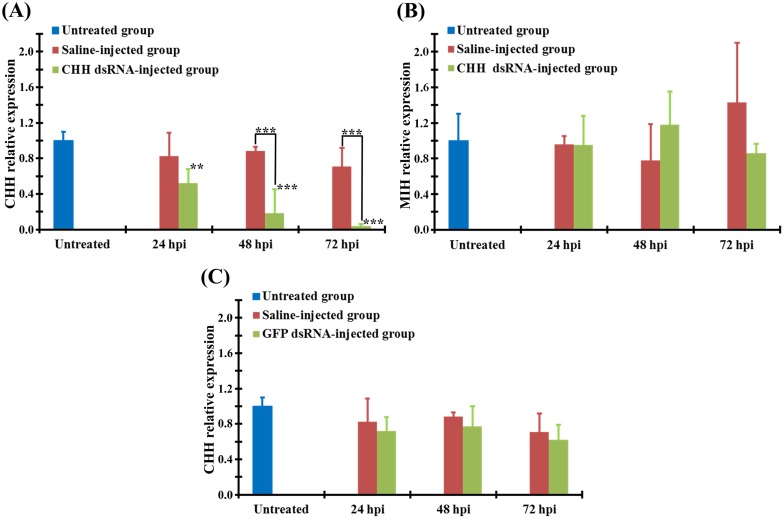
Effects of dsRNA treatment on transcript levels in the eyestalk ganglia of the crayfish *Procambarus clarkii*. Animals were separated into 3 groups: the untreated, saline-injected, and dsRNA-injected groups. Using a relative real-time qPCR, CHH (A, C) and MIH (B) transcript levels were quantified for the samples of the eyestalk ganglia harvested from the untreated animals (blue bar), and the saline-injected (crimson bar) and dsRNA-injected animals (green bar) at 24, 48, and 72 h post injection (hpi) of CHH dsRNA (A, B), GFP dsRNA (C), or saline (A, B, C). Transcript levels are normalized to a reference gene (*18S rRNA*) and expressed relative to the untreated levels. **, and *** represent significantly different from the untreated or the saline-injected values at the level of 0.01 and 0.005, respectively (n = 5 for each data).

Transcript levels in the eyestalk ganglia were quantified by a relative real-time PCR. Animals of the CHH dsRNA-injected (DSI) group exhibited a progressive decrease in CHH transcript levels over a period of 72 h post injection (hpi). Thus, compared to the those in untreated animals, CHH transcript levels in CHH DSI animals were significantly lower, 52.0 ± 15.8%, 18.5 ± 27.0%, and 4.0 ± 2.4% of the untreated value at 24, 48, and 72 hpi, respectively (Fig 1A). On the contrary, CHH transcript levels in the saline-injected (SAI) animals were relatively stable from 24 to 72 hpi, not significantly different from those in the untreated animals at any time point examined (82.4 ± 26.4%, 88.5 ± 4.7%, and 70.9 ± 21.1% of the untreated value at 24, 48, and 72 hpi, respectively), but were higher than those in the CHH DSI animals at each time point (with statistical significance being recorded at 48 and 72 hpi) (Fig 1A). These results clearly indicate that expression of CHH gene in the eyestalk ganglia was significantly knocked down by CHH dsRNA treatment.

Specificity of the CHH dsRNA treatment was tested by examining its effect on the transcript levels of a gene encoding another CHH family-peptide, molt-inhibiting hormone (MIH). Compare to the untreated levels, MIH transcript levels in the CHH DSI animals were not significantly different, 95.3 ± 32.8%, 117.8 ± 37.5%, and 86.3 ± 10.2% of the untreated value at 24, 48, and 72 hpi, respectively (Fig 1B). Similarly, MIH transcript levels in the SAI animals were not significantly different from the untreated levels (95.7 ± 9.7%, 77.7 ± 40.7%, and 143.4 ± 67.0% of the untreated value at 24, 48, and 72 hpi, respectively), nor were they significantly different from those in the CHH DSI group at any time point (Fig 1B). Specificity of the silencing effect of CHH dsRNA treatment on CHH was additionally tested by another dsRNA with a sequence unrelated to CHH–green fluorescence protein (GFP) dsRNA. CHH transcript levels in the GFP DSI or SAI animals were not significantly from those in the untreated animals (DSI: 72.0 ± 15.8%, 77.0 ± 23.0%, and 62.0 ± 17.0% of the untreated value; SAI: 82.4 ± 26.4%, 88.5 ± 4.7%, and 70.9 ± 21.1% of the untreated value, at 24, 48, and 72 hpi, respectively), nor were they significantly different from each other at any time point examined (Fig 1C).

### Effects of CHH dsRNA on the levels of CHH peptide in the sinus gland and hemolymph

**Fig 2:**
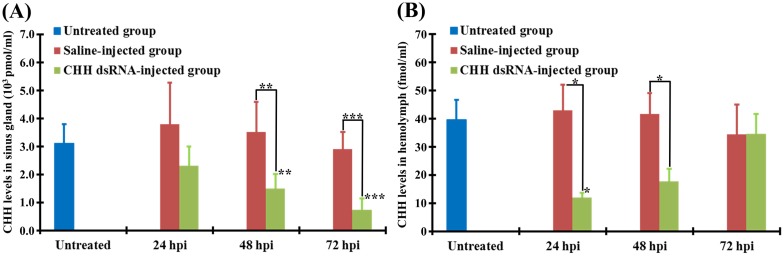
Effects of CHH dsRNA treatment on CHH peptide levels in the crayfish *Procambarus clarkii*. Animals were separated into 3 groups: the untreated, saline-injected, and dsRNA-injected groups. Using a CHH-specific sandwich ELISA, CHH peptide levels were quantified for the samples of the sinus gland (A) and hemolymph (B) harvested from the untreated animals (blue bar), and the saline-injected animals (crimson bar) and CHH dsRNA-injected animals (green bar) at 24, 48, and 72 h post injection (hpi)of dsRNA or saline. *,**, and *** represent significantly different from the untreated or saline-injected values at the level of 0.05, 0.01 and 0.005, respectively (n = 5 for each data).

CHH peptide levels in the sinus gland and hemolymph were measured by a sandwich ELISA. CHH DSI animals exhibited a progressive decrease in CHH peptide levels in the sinus gland over a period of 72 h, with a temporal profile similar to that of changes in transcript levels. Compared to the untreated levels (3.1 ± 0.7 x10^3^ pmole/mg protein), CHH levels in CHH DSI animals were lower at 24 hpi (2.3 ± 0.7 x10^3^ pmole/mg protein) and significantly lower at 48 and 72 hpi (1.5 ± 0.5 x10^3^ and 0.7 ± 0.4 x10^3^ pmole/mg protein, respectively) (Fig 2A). On the contrary, CHH peptide levels in the SAI animals remained relatively stable from 24 to 72 hpi (3.8 ± 1.5, 3.5 ± 1.1, 2.9 ± 0.6 x10^3^ pmole/mg protein, respectively), not significantly different from those in the untreated animals at any time point examined, but were significantly higher than those in the CHH DSI animals at 48 and 72 hpi (Fig 2A).

Compared to those of the untreated animals (39.5 ± 7.0 fmole/ml), CHH peptide levels in the hemolymph of the CHH DSI animals at 24 hpi (12.0 ± 1.6 fmole/ml) were significantly decreased, but were not significantly changed at 48 and 72 hpi (17.8 ± 4.4 and 34.7 ± 6.9 fmole/ml, respectively) (Fig 2B). CHH peptide levels in the SAI animals remained relatively stable from 24 to 72 hpi (42.9 ± 9.1, 41.7 ± 7.4, 34.4 ± 10.6 fmole/ml, respectively), not significantly different from those in the untreated animals at any time point examined, but significantly higher than those in the CHH DSI animals at 24 and 48 hpi (Fig 2B).

### Effects of CHH dsRNA on the levels of hemolymph glucose

**Fig 3:**
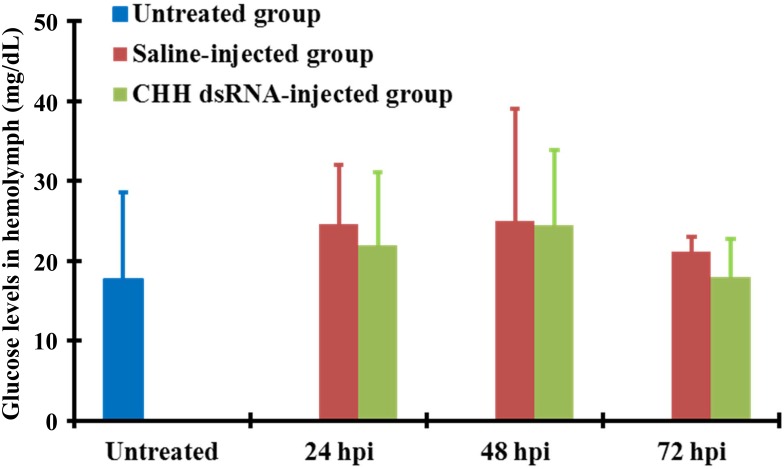
Effects of CHH dsRNA treatment on hemolymph glucose levels in the crayfish *Procambarus clarkii*. Animals were separated into 3 groups: the untreated, saline-injected, and dsRNA-injected groups. Using a glucose assay, glucose levels were quantified for the hemolymph samples harvested from the untreated animals (blue bar), and the saline-injected animals (crimson bar) and CHH dsRNA-injected animals (green bar) at 24, 48, and 72 h post injection (hpi) of dsRNA or saline (n = 5 for each data).

There was no significant change in hemolymph glucose levels for DSI or SAI animals from 24 to 72 hpi (DSI: 21.9 ± 9.2, 24.4 ± 9.4, and 17.9 ± 4.8 mg/dL, respectively; SAI: 24.5 ± 7.5, 24.9 ± 14.1, and 21.1 ± 1.9 mg/dL, respectively), nor were the glucose levels significantly different between the 2 groups of animals at each time point (Fig 3). Hemolymph glucose levels for DSI or SAI animals at any time point examined were not significantly different from the untreated levels (Fig 3).

### Effects of CHH dsRNA on the levels of tissue metabolites

**Fig 4:**
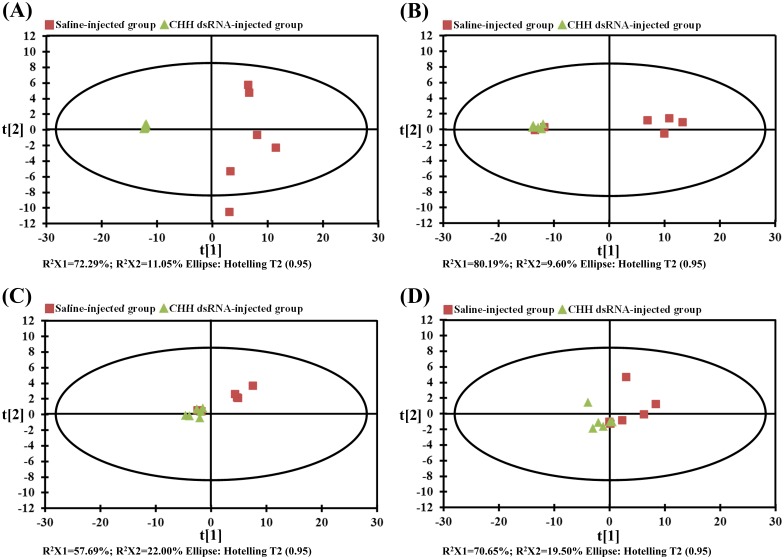
Principal component analysis plot of tissue samples of the crayfish *Procambarus clarkii* showing differential metabolic profiles between saline-injected and CHH dsRNA-injected groups. A, B: the muscle at 24 and 48 hpi, respectively; C, D: the hepatopancreas at 24 and 48 hpi, respectively. Triangle: CHH dsRNA injected group; Square: saline-injected group. Each symbol represents the metabolome of a single sample along two principal components.

Tissue metabolites detected, quantified, and found significantly different in metabolite levels between SAI and CHH DSI groups were listed in supplementary tables (S2 and S3 Tables), which includes 149 and 181 metabolites from the muscle at 24 and 48 hpi, respectively, and 24 and 12 metabolites from the hepatopancreas at 24 and 48 hpi, respectively. Levels of these metabolites in the untreated animals are also given for reference and they are not significantly different from those of the SAI group (S2 and S3 Tables). Principal component analysis (PCA) of the metabolites in the muscle data show a clear separation between SAI versus CHH dsRNA groups at 24 hpi (Fig 4A; combined contribution of the first two PCs explained 83.3% of the total variation), while the separation became less distinguishable at 48 hpi (Fig 4B; combined contribution of the first two PCs explained 89.8% of the total variation). PCA for the metabolites in the hepatopancreas, at both 24 and 48 hpi, show that the cluster of CHH DSI group was somewhat separable from that of SAI group but the separations were less clear-cut than those in the muscle (Fig 4C and 4D; combined contribution of the first two PCs explained 79.7% and 90.2% of the total variation for 24 and 48 hpi, respectively).

Levels of the metabolites that are closely related to carbohydrate and energy metabolism in the tissues of DSI and SAI animals are presented and statistically compared in Tables 1 and 2 and described below. In the muscle, 24 h after CHH dsRNA treatment, when hemolymph CHH levels were significantly decreased compared to those of the untreated and the SAI groups, levels of the reduced form of nicotinamide adenine dinucleotide (NADH), as well as those of its oxidized form (NAD^+^), were significantly reduced, with ratio of NADH/NAD^+^ being significantly increased. Levels of 2 precursors to NAD^+^ (quinolinate, deamino-NAD^+^) were also significantly decreased (Table 1). In addition, levels of ATP and ADP were significantly decreased, with the ratio of ATP/ADP remained relatively constant; likewise, levels of AMP were significantly decreased (Table 1). Regarding glycolytic metabolites, levels of 2-phosphoglycerate, pyruvate, and lactate were significantly decreased (Table 1). Levels of 2 monosaccharides (fructose and galactose) and 2 disaccharides (sucrose and lactose) were also significantly decreased, while the decrease in levels of glucose was not significant. Tricarboxylic acid (TCA) cycle was also affected, with levels of oxaloacetate, citrate, and isocitrate significantly increased, but those of malate significantly decreased. Moreover, levels of 2 ketone bodies (β-hydroxybutyrate and acetoacetate) were also significantly reduced.

**Table 1:** Effects of CHH dsRNA treatment on levels of metabolites related to energy and carbohydrate metabolism in the muscle of the crayfish *Procambarus clarkii*.

			2-Phosphoglycerate	Acetaldehyde	Acetate	Acetoacetate	ADP	AMP	ATP	ATP/ADP	Citrate	Cystathionine	Cysteine
24 hpi	Saline-injected group (SAI)	Mean	0.00343	0.00339	0.01676	0.00436	0.00368	0.00464	0.00361	0.98588	0.86102	1.64975	1.52669
		Std	0.00073	0.00169	0.0112	0.0019	0.00126	0.00136	0.00127	0.09011	0.02227	0.17732	0.35846
	CHH dsRNA-injected group (DSI)	Mean	0.00035	0.00037	0.00148	0.00051	0.00055	0.00067	0.00059	1.07124	0.95117	1.92118	1.90964
		Std	0.00013	0.00009	0.00065	0.00023	0.00005	0.00015	0.00005	0.02225	0.00534	0.00805	0.01002
	p value		0.00063	0.00714	0.02045	0.00408	0.00173	0.00079	0.00213	0.06805	0.00333	0.01327	0.07525
	% SAI^a^		10.1	11	8.8	11.6	15.1	14.5	16.5	108.7	110.5	116.5	125.1
48 hpi	Saline-injected group (SAI)	Mean	0.00274	0.00266	0.01224	0.00276	0.00317	0.00383	0.00312	1.00136	0.90777	1.82464	1.79923
		Std	0.00176	0.0017	0.00754	0.00164	0.00201	0.00235	0.00199	0.07501	0.03577	0.07725	0.08638
	CHH dsRNA-injected group (DSI)	Mean	0.00045	0.00043	0.00194	0.00088	0.00051	0.00061	0.00054	1.05425	0.94987	1.90972	1.89782
		Std	0.00026	0.0002	0.00084	0.00071	0.00014	0.00021	0.00014	0.05821	0.00447	0.02842	0.02617
	p value		0.02396	0.02343	0.02012	0.03724	0.0225	0.02015	0.02443	0.18482	0.03422	0.04219	0.03728
	% SAI^a^		16.4	16.2	15.8	32.1	16	15.9	17.2	105.3	104.6	104.7	105.5
			deamino-NAD^+^	Fructose	Fumarate	Galactose	Glucose	Isocitrate	Lactate	Lactose	Malate	Methionine	NAD^+^
24 hpi	Saline-injected group (SAI)	Mean	0.00649	0.0088	0.00052	0.0065	0.00816	0.86257	0.0036	0.01269	0.02338	0.04774	0.01047
		Std	0.00309	0.00108	0.00045	0.00089	0.00375	0.0262	0.00143	0.00341	0.00758	0.01984	0.00385
	CHH dsRNA-injected group (DSI)	Mean	0.00115	0.00112	0.00018	0.00093	0.0048	0.96713	0.0004	0.00286	0.00586	0.00524	0.00185
		Std	0.0001	0.00048	0.00004	0.00039	0.00087	0.00372	0.00012	0.00052	0.00088	0.00198	0.00013
	p value		0.008194	0.00002	0.12264	0.00005	0.11601	0.00387	0.00266	0.00278	0.00647	0.02306	0.00273
	% SAI^a^		17.8	12.7	35.1	14.3	58.8	112.1	11	22.5	25.1	11	17.6
48 hpi	Saline-injected group (SAI)	Mean	0.00603	0.00684	0.00058	0.00518	0.00721	0.91471	0.00308	0.01078	0.01753	0.02819	0.00947
		Std	0.00392	0.00407	0.00062	0.00294	0.00185	0.04086	0.00213	0.00603	0.00219	0.01556	0.006
	CHH dsRNA-injected group (DSI) group	Mean	0.00107	0.002	0.00017	0.00153	0.00794	0.96577	0.00041	0.00499	0.00922	0.00775	0.00171
		Std	0.0002	0.00182	0.00005	0.00124	0.00622	0.00411	0.00019	0.00419	0.00897	0.00438	0.00026
	p value		0.02665	0.03207	0.16497	0.02716	0.76153	0.0279	0.02746	0.05472	0.03668	0.02242	0.02478
	% SAI^a^		17.7	29.2	29.4	29.5	110.1	105.6	13.2	46.3	52.6	27.5	18.1
			NADH	NADH/NAD^+^	Oxaloacetate	Pyruvate	Quinolinate	Serine	Succinate	Sucrose	cis-Aconitate	α-Ketobutyrate	β-Hydroxybutyrate
24 hpi	Saline-injected group (SAI)	Mean	0.01958	1.81372	0.00236	0.0011	0.00488	0.00411	0.00921	0.00669	0.83467	0.00366	0.03871
		Std	0.00537	0.28467	0.00092	0.00082	0.002	0.00117	0.00697	0.00149	0.0363	0.00084	0.01524
	CHH dsRNA-injected group (DSI)	Mean	0.00729	3.95632	0.00405	0.00014	0.00105	0.00056	0.00121	0.00096	0.95381	0.00157	0.01281
		Std	0.00076	0.41415	0.00092	0.00008	0.00037	0.00035	0.00026	0.00036	0.00462	0.00022	0.00057
	p value		0.00658	3.24E-07	0.02317	0.0341	0.00509	0.00189	0.06215	0.00077	0.16147	0.00442	0.0088
	% SAI^a^		37.2	218.1	171.7	12.5	21.6	13.5	13.2	14.3	114.3	42.8	33.1
48 hpi	Saline-injected group (SAI)	Mean	0.0209	1.57793	0.0031	0.00082	0.00461	0.00349	0.00381	0.00528	0.89439	0.0027	0.02795
		Std	0.00148	0.10214	0.00081	0.00063	0.00295	0.00203	0.00191	0.00294	0.04509	0.00087	0.00352
	CHH dsRNA-injected group (DSI)	Mean	0.01119	4.19374	0.00661	0.00024	0.0009	0.00076	0.00172	0.00161	0.93489	0.00163	0.01342
		Std	0.01075	0.71874	0.0052	0.00021	0.0005	0.00063	0.0014	0.00134	0.05014	0.0003	0.00253
	p value		0.03828	0.00005	0.09931	0.07367	0.02701	0.02045	0.05068	0.02666	0.14013	0.0275	0.00102
	% SAI^a^		53.5	265.8	213.5	29.1	19.5	21.7	45.1	30.5	104.5	60.2	48

^a^ % SAI = (CHH DSI value/SAI value) x 100.

n = 6 for each data.

**Table 2:** Effects of CHH dsRNA treatment on levels of metabolites related to energy and carbohydrate metabolism in the hepatopancreas of the crayfish *Procambarus clarkii*.

			2-Phosphoglycerate	Acetaldehyde	Acetate	Acetoacetate	ADP	AMP	ATP	ATP/ADP	Citrate
24 hpi	Saline-injected group (SAI)	Mean	1.23	0.00131	0.00498	0.0026	0.0024	0.0137	0.0022	0.95707	0.0213
		Std	1.06405	0.0005	0.0007	0.00053	0.00211	0.01575	0.00195	0.06961	0.00681
	CHH dsRNA-injected group (DSI)	Mean	1.84987	0.00079	0.00412	0.00247	0.00214	0.01213	0.00198	0.95029	0.02479
		Std	0.01851	0.00023	0.00059	0.00076	0.00122	0.00754	0.0011	0.06176	0.00697
	p value		0.28186	0.02157	0.02756	0.72378	0.78838	0.8154	0.80171	0.87844	0.56125
	% SAI^a^		150.4	60.4	82.7	94.9	89.2	88.5	89.9	99.3	116.4
48 hpi	Saline-injected group (SAI)	Mean	1.83896	0.00102	0.00458	0.00331	0.00255	0.01344	0.00239	0.94588	0.02019
		Std	0.04985	0.00054	0.00038	0.00078	0.0016	0.0076	0.00146	0.04946	0.00148
	CHH dsRNA-injected group (DSI)	Mean	1.86875	0.00099	0.00499	0.00474	0.00216	0.01174	0.00204	0.96204	0.01844
		Std	0.01007	0.00057	0.00142	0.00384	0.00111	0.00732	0.00101	0.05158	0.00278
	p value		0.25824	0.9018	0.49009	0.37256	0.67888	0.72922	0.69084	0.6246	0.18055
	% SAI^a^		101.6	96.2	108.9	143.2	84.4	87.3	85.4	101.7	91.3
			Fructose	Fumarate	Galactose	Glucose	Isocitrate	Lactate	Lactose	Malate	NAD^+^
24 hpi	Saline-injected group (SAI)	Mean	1.52608	0.00015	0.69862	1.83434	0.38443	0.00318	1.90983	0.01394	0.00905
		Std	1.31517	0.00005	0.60071	1.57217	0.50044	0.00271	1.64139	0.00258	0.00578
	CHH dsRNA-injected group (DSI)	Mean	2.26947	0.00013	1.01698	2.77756	0.37728	0.0026	2.81394	0.01538	0.00719
		Std	0.00634	0.00003	0.00627	0.03435	0.46156	0.00143	0.01891	0.00617	0.00301
	p value		0.29468	0.43559	0.20367	0.15667	0.98354	0.64652	0.30586	0.63267	0.50686
	% SAI^a^		148.7	87.8	145.6	151.4	98.1	81.8	147.3	110.4	79.4
48 hpi	Saline-injected group (SAI)	Mean	1.51985	0.00016	0.86221	2.29038	0.38168	0.00376	2.17322	0.01445	0.00676
		Std	1.28494	0.00009	0.40964	0.63201	0.46777	0.00193	1.10769	0.0018	0.00265
	CHH dsRNA-injected group (DSI) group	Mean	2.28056	0.00016	1.02053	2.80172	0.36094	0.00306	2.51827	0.01367	0.00777
		Std	0.00641	0.00007	0.00376	0.0251	0.45123	0.00101	0.77801	0.0023	0.00283
	p value		0.41299	0.91008	0.38726	0.2504	0.9525	0.49503	0.76795	0.52557	0.6201
	% SAI^a^		150.1	96.6	118.4	122.3	94.6	81.4	115.9	94.6	115
			NADH	NADH/NAD^+^	Oxaloacetate	Pyruvate	Succinate	Sucrose	cis-Aconitate	β-Hydroxybutyrate	
24 hpi	Saline-injected group (SAI)	Mean	0.03327	3.9597	0.63987	0.00085	0.00448	2.53967	0.31075	0.01883	
		Std	0.01823	0.68821	0.54205	0.0002	0.00089	2.19471	0.52572	0.00446	
	CHH dsRNA-injected group (DSI)	Mean	0.02699	3.93722	0.95191	0.00062	0.00436	3.87046	0.24418	0.02559	
		Std	0.00965	0.57783	0.00922	0.00017	0.00149	0.01908	0.43899	0.0091	
	p value		0.48237	0.96332	0.1717	0.03826	0.86702	0.35288	0.83539	0.14069	
	% SAI^a^		81.1	99.4	148.8	72.6	97.3	152.4	78.6	135.9	
48 hpi	Saline-injected group (SAI)	Mean	0.02525	3.72475	0.94828	0.00073	0.00458	2.49687	0.23749	0.01872	
		Std	0.00995	0.61928	0.01216	0.00035	0.00085	2.13694	0.45763	0.00048	
	CHH dsRNA-injected group (DSI)	Mean	0.0275	3.67413	0.95547	0.00076	0.00483	3.92309	0.27263	0.01908	
		Std	0.00623	0.52807	0.00803	0.00041	0.00197	0.02581	0.45606	0.00434	
	p value		0.71723	0.90518	0.2498	0.87741	0.76946	0.36712	0.90617	0.83544	
	% SAI^a^		108.9	98.6	100.8	104.6	105.4	157.1	114.8	101.9	

^a^ % SAI = (CHH DSI value/SAI value) x 100.

n = 6 for each data.

Metabolic profile in the muscle of CHH DSI group at 48 hpi was similar to that at 24 hpi (Table 1). Levels of the majority of the metabolites were similarly significantly changed at 48 hpi (but in general with weaker levels of statistical significance), except those of several metabolites (pyruvate, lactose, and oxaloacetate) that became not statistically significantly changed (compared to those of SAI group) (Table 1).

Effects of CHH dsRNA treatment on the hepatopancreas were relatively mild and short-lasting (Table 2), compared to those on the muscle (Table 1). At 24 hpi, levels of ATP and ADP, as well as the ratio of ATP/ADP, remained relatively constant; so did levels of NADH and NAD^+^, and the ratio of NADH/NAD^+^ (Table 2). Levels of fructose, galactose, sucrose, and lactose were slightly increased (although not significantly) (Table 2). Levels of 2-phosphoglycerate was increased (not significantly) and those of pyruvate, acetaldehyde, and acetate significantly decreased (Table 2). As for the TCA cycle intermediates, only levels of oxaloacetate were increased (although not significantly), while those of the rest of intermediates remained relatively constant (Table 2). Levels of the 2 ketone bodies were either remained relatively constant (acetoacetate) or slightly increased (β-hydroxybutyrate).

Metabolic profile in the hepatopancreas of CHH DSI animals at 48 hpi is essentially not different from that of SAI animals. None of the metabolites was significantly different in levels between CHH DSI and SAI animals (Table 2), including pyruvate, acetaldehyde, and acetate that were significantly decreased at 24 hpi.

## Discussion

To evaluate metabolic effects of crustacean hyperglycemic hormone on its targets, a double-stranded RNA-based gene silencing method was developed and validated. Resulting data indicate that CHH dsRNA injected *in vivo* effectively silenced CHH expression in the crayfish *P*. *clarkii* at the levels of CHH transcript expressed in the eyestalk ganglia, as well as those of CHH protein in the sinus gland and hemolymph, at least from 24 to 48 h after dsRNA treatment. Silencing specificity was demonstrated by a lack of effect of the CHH dsRNA on the expression of a gene encoding another CHH-family peptide–MIH, and additionally confirmed by a treatment of GFP dsRNA, which did not affect CHH gene expression.

**Fig 5:**
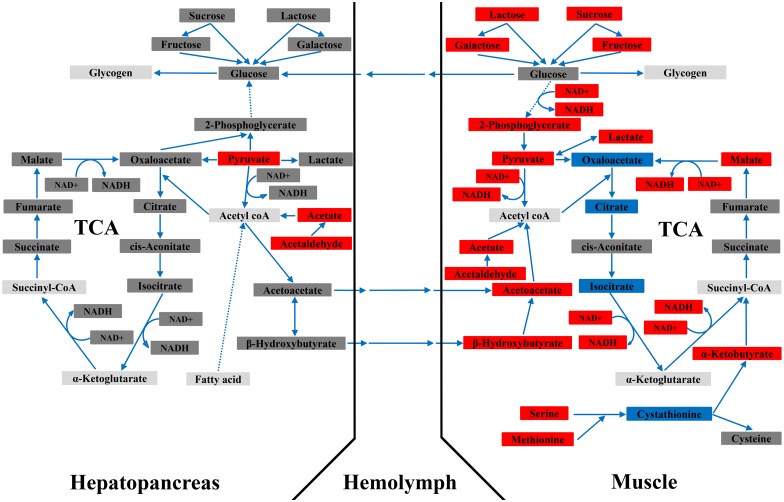
Metabolic profiles of the muscle and hepatopancreas of the crayfish *Procambarus clarkii* 24 h after CHH dsRNA treatment. Red rectangle: significantly decreased from saline-injected levels; blue rectangle: significantly increased from saline-injected levels; dark gray rectangle: not significantly changed; light gray rectangle: not detected. Data obtained at 24 hpi (as listed in Tables 1 and 2) were used for constructing the metabolic networks relating to carbohydrate and energy metabolism. Dotted lines indicate multiple metabolic steps are involved that are not individually specified.

Metabolic data reported in the present study clearly show that silencing of CHH gene expression significantly altered metabolic profiles of 2 CHH target tissues–the muscle and hepatopancreas, with the effects being metabolically manifested in tissue-specific but complementary manners (see below and Fig 5). PCA score plots indicate that the impacts were more pronounced, especially at 24 h after dsRNA treatment, for the muscle than for the hepatopancreas. Importantly, the observed metabolic changes suggest a much wider array of effects of CHH than previously realized [12].

Both tissues were more significantly impacted by CHH dsRNA treatment at 24 hpi, with the metabolic status at 48 hpi being reversing towards the saline-treated levels. Hence metabolic profiles for each tissue are discussed below based on changes at 24 h post CHH dsRNA treatment (see Fig 5). Central to these metabolic changes in the muscle are decreased levels of NAD^+^ and adenine ribonucleotides (AMP and ADP) (Table 1). It is well-established that glycolytic flux is dependent on a continuous supply of NAD^+^ [44]. Hence the observed inhibition of glycolysis (as evidenced by significantly lower levels of 2-phosphoglycerate, pyruvate, and lactate) (Fig 5) is at least in part due to low NAD^+^ availability. Consequently, glucose, instead of entering glycolysis, was presumably diverted for glycogen synthesis either intramuscularly or, perhaps more likely, in the hepatopancreas, after it was transported to this tissue (Fig 5). The suggested transport of glucose from the muscle to the hepatopancreas would therefore explain why hemolymph glucose levels in the CHH DSI animals, compared to those in the SAI animals, were not decreased in response to significantly lower levels of hemolymph CHH. Regarding other carbohydrates, significantly lower levels of sucrose, lactose, fructose, and galactose would most likely reflect contribution of these sugars to glycogen synthesis via glucose (Fig 5). Moreover, TCA cycle, where NAD^+^ is reduced to NADH in 3 of the reactions, was apparently inhibited (Fig 5). The inhibition was likely also due to low availability of NAD^+^, which inhibits the three oxidation steps for which NAD^+^ is the cofactor (Lehninger et al., 1993); the reactions of TCA cycle that carry isocitrate through to oxaloacetate were inhibited, with levels of all the detected intermediates being decreased (Fig 5). Thus, diminished levels of NAD^+^ (and high NADH/NAD^+^ ratio), observed when CHH gene expression was silenced, is responsible at least partly for reducing TCA cycle flux. The flux was, however, partially rescued by (1) the 2 ketone bodies, β-hydroxybutyrate and acetoacetate (presumably derived from the hepatopancreas; see below) through their conversion to acetyl CoA, which in turn enters the cycle at the point of citrate formation and (2) by an anaplerotic reaction that feeds into the TCA cycle at the point of succinyl-CoA (from α-ketobutyrate that was derived from serine and methionine) (Fig 5). Finally, another prominent feature observed when CHH gene was silenced is severely diminished levels of ATP, which is closely related to the metabolic changes mentioned above. Thus, significantly lower levels of NADH, due mainly to a negatively impacted TCA cycle flux, is one of the factors that limited production of ATP through oxidative phosphorylation. Another factor that may lead to significantly lower levels of ATP would be low availability of its mono- and di-phosphate precursors–AMP and ADP (Table 1).

Thus, metabolic profile of the muscle under the condition of silenced CHH expression was characterized by significantly reduced synthesis of NAD^+^ and adenine ribonucleotides, lower rate of utilization of carbohydrates (glucose and other sugars) through glycolysis, and a negatively impacted (but partially rescued) TCA cycle, and greatly diminished supply of ATP.

Several earlier studies had suggested that CHH stimulates glycolytic flux [28, 29], although such a proposition has never been directly tested. Our observation that glycolytic flux was significantly negatively impacted by silencing expression of CHH gene provides the first piece of evidence indicating that CHH indeed exerts stimulatory effects on glycolysis. As discussed above, metabolic effects of CHH in the muscle are not limited to glycolysis, and regulation of synthesis of NAD^+^ and adenine ribonucleotides is central to the metabolic roles played by CHH. In this regard, it is interesting to note that, 5-phsophoribosyl 1-pyrophosphae (PRPP) synthase is a tightly regulated enzyme whose product (PRPP) is a precursor for the synthesis of not only ribonucleotides but also for the synthesis of NAD^+^ [45, 46]. Thus, it is entirely possible that CHH could have diverse metabolic effects by regulating both biosynthetic pathways (NAD^+^ and adenine ribonucleotides) via affecting the activity of certain critical enzymes such as PRPP synthase.

In light of the metabolic effects of CHH suggested by the present study, it is relevant to discuss these data with findings reported by earlier studies [40, 47]. First, it has been shown that white spot syndrome virus (WSSV) activated CHH gene expression and enhanced release of CHH from the sinus glands in the crayfish *Procambarus clarkii*, resulting in an acute onset of significant increase, commencing as early as 3 h post infection, in hemolymph CHH levels that lasted for at least 2 days [40]. In other line of studies, an invertebrate Warburg effect, characterized by up-regulation of several metabolic pathways, including glycolysis, the pentose phosphate pathway, ribonucleotide biosynthesis, glutaminolysis and amino acid biosynthesis, was observed in the hosts (*Litopenaeus vannamei*) at the viral genome replication stage (12 h after being infected by WSSV) [47]. Taken together, these results and those presented in this study strongly indicate that CHH released by WSSV infection is responsible for inducing at least certain aspects of the WSSV-induced Warburg effect, including enhanced glycolysis, increased adenine ribonucleotide synthesis.

Compared to the muscle, the hepatopancreas exhibited less drastic changes in response to silencing of CHH gene expression. Both ratios of NADH/NAD^+^ and ATP/ADP were not significantly affected, nor were the levels of each metabolite. Accumulation of fructose, galactose, sucrose, and lactose probably reflected an increased uptake by the hepatopancreas of these sugars, which were likely to be converted into glycogen via glucose (Fig 5). Glycolysis in the hepatopancreas was apparently also inhibited, as levels of pyruvate and lactate were decreased (Fig 5). Given the observation that levels of pyruvate and lactate were lower, increases (although not significant) in levels of 2-phosphoglycerate (a common intermediate for both glycolysis and gluconeogenesis) and glucose (Fig 5) suggest that rate of gluconeogenesis was elevated. Moreover, levels of β-hydroxybutyrate (a ketone body) were increased (Fig 5), an indication of excess acetyl CoA exceeding the capacity of TCA cycle, and presumably reflected enhanced β-oxidation of fatty acids under low glucose oxidation through glycolysis [48]. In addition, the 2 ketone bodies (β-hydroxybutyrate and acetoacetate) were most likely to be transported to the muscle, where they were converted into acetyl CoA and subsequently enter TCA cycle via formation of citrate (Fig 5).

Thus, metabolic status of the hepatopancreas under the condition of silenced CHH expression, in many ways different from that of the muscle, was characterized by unaffected supply of ATP, lower rate of utilization of carbohydrates (inhibited glycolysis and probably stimulated glycogen synthesis), and increased synthesis of ketone bodies (that were likely to be transported to the muscle) presumably via enhanced β-oxidation of fatty acids (Fig 5).

The metabolic responses of the muscle and hepatopancreas to silencing of CHH expression were obviously different but complementary. It has been showed that eyestalk ablation significantly decreased transcript levels of glycogen phosphorylase (GP) and increased those of glycogen synthase (GS) in the hepatopancreas, but not in muscle, in the kuruma prawn *Marsupenaeus japonicas* [23]. Conversely, transcript levels of GP were significantly increased and those of GS decreased, after hepatopancreatic tissues were treated *ex vivo* with recombinant CHH [23]. Thus, these data not only support our suggestion that with silenced CHH expression glycogen synthesis would be increased in the hepatopancreas, but also indicate that muscle and hepatopancreas were differentially regulated in terms of glycogen metabolism by CHH, highlighting the point we made that CHH regulates its target tissues in a tissue-specific manner. On the other hand, the differential responses after CHH gene silencing were complementary in that glucose was suggested to be transported from the muscle to the hepatopancreas for glycogen synthesis and that acetyl CoA synthesized via β-oxidation of fatty acids in the hepatopancreas was used for production of ketone bodies to be transported to the muscle (Fig 5).

In summary, a double-stranded RNA-based method was developed and validated for effective and specific silencing of CHH gene. When applied *in vivo* to the experimental animals (*Procambarus clarkii*), it allowed us to clearly reveal the differential and complementary effects of silencing CHH gene expression on two CHH target tissues. These data imply that CHH has a range of effects on these tissues much more diverse than previously known. Finally, but not least importantly, combined data of this and other previous studies [40, 47] suggest that the WSSC-released CHH is at least partly responsible for inducing the invertebrate Warburg effect that was manifested in infected hosts and thought to be beneficial for viral replication.

## Supporting information

S1 Fig Confirmation of integrity of the double-stranded RNA. CHH (A) or GFP (B) dsRNA produced using *in vitro* transcription reactions were separated by 2% agarose electrophoresis. M: RNA markers. Positions of 100-bp and 200-bp makers are labeled.
(TIF)

S1 Table Sequences of primers used in the present study. (PDF)

S2 Table List of metabolites in the muscle of the crayfish *Procambarus clarkii* whose levels were significantly different between the saline-injected and CHH dsRNA-injected animals at 24 and 48h post injection (hpi). Data are expressed as mean ± standard deviation (n = 6 for each data). ^a^ Levels of the metabolites in the untreated animals are also included for reference.
(PDF)

S3 Table List of metabolites in the hepatopancreas of the crayfish *Procambarus clarkii* whose levels were significantly different between the saline-injected and CHH dsRNA-injected animals at 24 and 48h post injection (hpi). Data are expressed as mean ± standard deviation (n = 6 for each data). ^a^ Levels of the metabolites in the untreated animals are also included for reference.
(PDF)
