# Supplementary figures and images for: Differential effects of silencing crustacean hyperglycemic hormone gene expression on the metabolic profiles of the muscle and hepatopancreas in the crayfish Procambarus clarkii
Source: PLoS One. 2017 Feb 16;12(2):e0172557. doi: 10.1371/journal.pone.0172557 (PMC5313166; doi:10.1371/journal.pone.0172557)

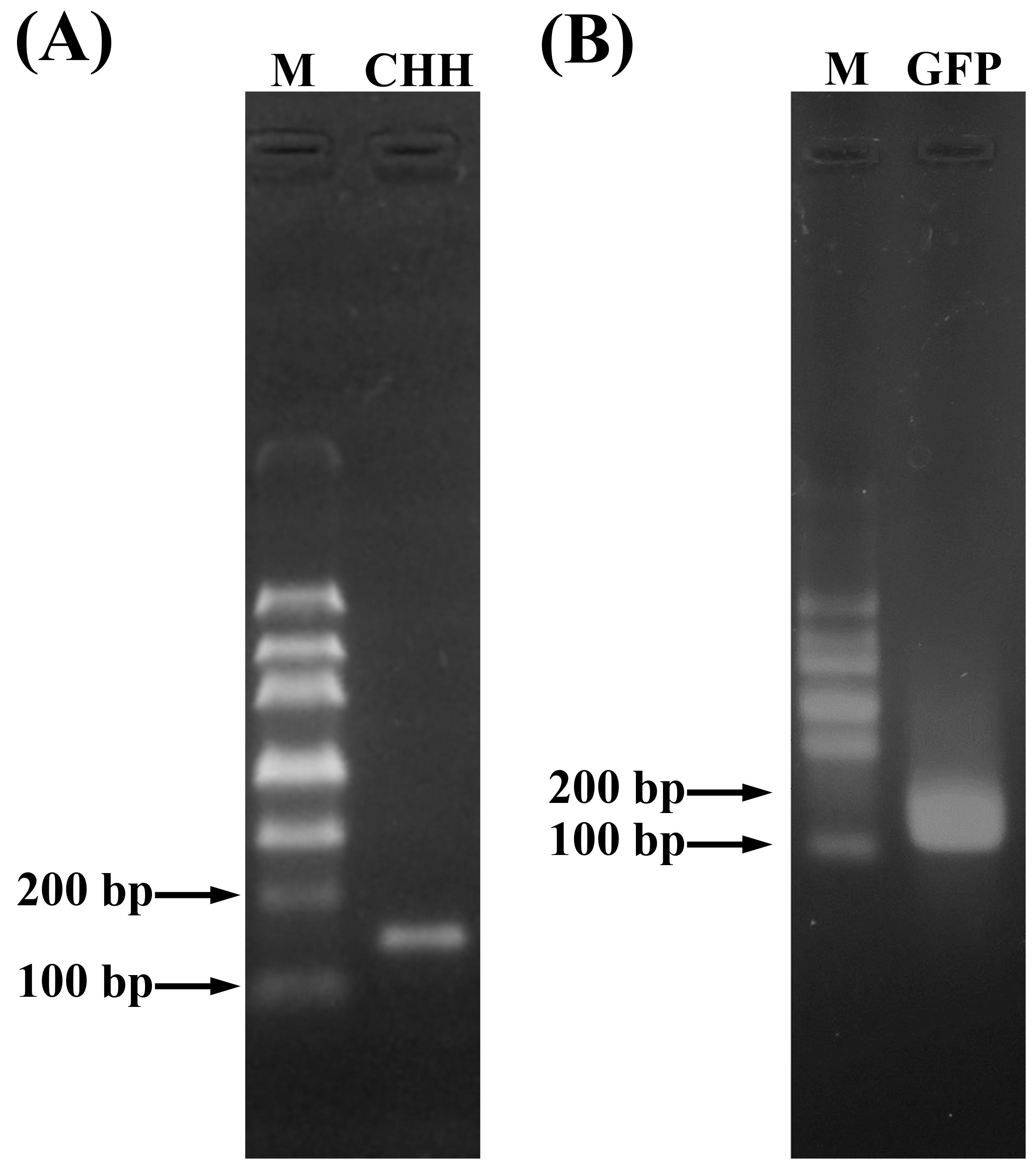

Supplement: S1 Fig — CHH (A) or GFP (B) dsRNA produced using in vitro transcription reactions were separated by 2% agarose electrophoresis. M: RNA markers. Positions of 100-bp and 200-bp makers are labeled. (TIF) [file pone.0172557.s001.tif]
