# Supplementary material for: Differential effects of silencing crustacean hyperglycemic hormone gene expression on the metabolic profiles of the muscle and hepatopancreas in the crayfish Procambarus clarkii
Source: PLoS One. 2017 Feb 16;12(2):e0172557. doi: 10.1371/journal.pone.0172557 (PMC5313166; doi:10.1371/journal.pone.0172557)
Supplement: S1 Table — (PDF) [file pone.0172557.s002.pdf]

Primers	Forward/Reverse	Sequence (5'-3')	Accession / Plasmid number
T7-CHH1-352-375-F	F	GGATCCTAATACGACTCACTATAGGGACCGAGTGTGTGAAGATTGTTAC	AB027291
CHH1-472-492-R	R	AATGTACTCGTCAACAACGTC	
CHH1-352-375-F	F	GACCGAGTGTGTGAAGATTGTTAC	
T7-CHH1-472-492-R	R	GGATCCTAATACGACTCACTATAGGAATGTACTCGTCAACAACGTC	
CHHe2F	F	AACCTCTCAGCTTCCTCTCCCAAG	
SG-R-400-427	R	CATAGCAGTTTTGTCTGCAGGTGGTGGC	
MIH-F	F	AGATATGTCTTCGAGGAA	KY307849
MIH-R	R	GCGTCCAGCGTTAAGAAT	
18s-F-1280	F	TGGTGCATGGCCGTTCTTA	AF436001
18s-R-1360	R	AATTGCTGGAGATCCGTCGAC	
F-T7-GFP-P140-ds	F	GGATCCTAATACGACTCACTATAGGAAGGACCATGTGGTCTCTC	Plasmid 1649 (Addgene)
GFP-R-181-201-P144	R	GATGGAAGCGTTCAACTAGC	
GFP-F-58-78-P144	F	AAGGACCATGTGGTCTCTC	
R-T7-GFP-P140-ds	R	GGATCCTAATACGACTCACTATAGGGATGGAAGCGTTCAACTAGC	
